# NMR studies of group 8 metallodrugs: ^187^Os-enriched organo-osmium half-sandwich anticancer complex†

**DOI:** 10.1039/d1dt02213j

**Published:** 2021-08-24

**Authors:** Russell J. Needham, Ivan Prokes, Abraha Habtemariam, Isolda Romero-Canelón, Guy J. Clarkson, Peter J. Sadler

**Affiliations:** Department of Chemistry, University of Warwick Gibbet Hill Road Coventry CV4 7AL UK P.J.Sadler@warwick.ac.uk

## Abstract

We report the synthesis of the organo-osmium anticancer complex [Os(η^6^-*p*-cym)(*N*,*N*-azpy-NMe_2_)Br]PF_6_ (**1**) containing natural abundance ^187^Os (1.96%), and isotopically-enriched (98%) [^187^Os]-**1**. Complex **1** and [^187^Os]-**1** contain a π-bonded *para*-cymene (*p*-cym), a chelated 4-(2-pyridylazo)-*N*,*N*-dimethylaniline (azpy-NMe_2_), and a monodentate bromide as ligands. The X-ray crystal structure of **1** confirmed its half-sandwich ‘piano-stool’ configuration. Complex **1** is a member of a family of potent anticancer complexes, and exhibits sub-micromolar activity against A2780 human ovarian cancer cells (IC_50_ = 0.40 μM). Complex [^187^Os]-**1** was analysed by high-resolution ESI-MS, 1D ^1^H and ^13^C NMR, and 2D ^1^H COSY, ^13^C–^1^H HMQC, and ^1^H–^187^Os HMBC NMR spectroscopy. Couplings of ^1^H and ^13^C nuclei from the azpy/*p*-cym ligands to ^187^Os were observed with *J*-couplings (^1^*J* to ^4^*J*) ranging between 0.6–8.0 Hz. The ^187^Os chemical shift of [^187^Os]-**1** (−4671.3 ppm, determined by 2D ^1^H–^187^Os HMBC NMR) is discussed in relation to the range of values reported for related Os(ii) arene and cyclopentadienyl complexes (−2000 to −5200 ppm).

## Abstract 2

Synthesis of this ^187^Os-enriched organo-osmium azopyridine anticancer complex, has allowed determination of ^187^Os NMR J-couplings to ligand ^1^H and ^13^C, and the ^187^Os chemical shift. The complex was also characterised by X-ray crystallography and MS.

## Graphical abstract

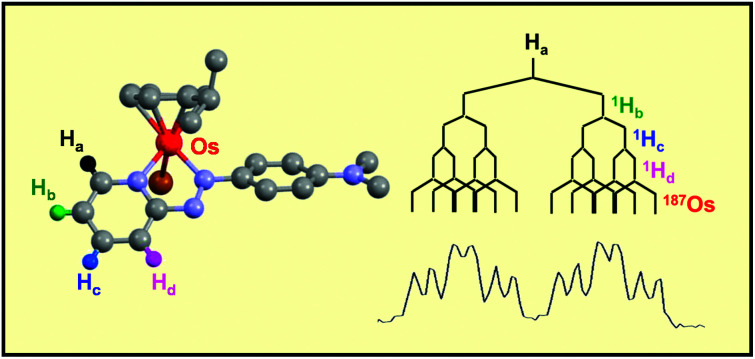

## Introduction

There is much current interest in the design of transition metal anticancer complexes.^1–4^ Progress depends on the elucidation of structure–activity relationships and their mechanisms of action, depends on the identification of their pharmacophores, the active species. Although some metal complexes are relatively inert (*e.g.* low-spin d^6^ complexes), many are pro-drugs which will undergo ligand exchange and redox reactions before reaching the target site. NMR is a potentially powerful method for investigating metallodrug speciation, since studies can be carried out in solution under physiologically relevant conditions, and information on the thermodynamics (equilibria) and kinetics (dynamics) of ligand exchange reactions can often be obtained. Direct observation of NMR resonances for the metals themselves (heteronuclear NMR) is potentially very informative, but often difficult to achieve.

We focus here on group 8 transition metals, Fe, Ru and Os. In particular two Ru(iii) complexes, NAMI-A ([*trans*-RuCl_4_(DMSO-*S*)(Im)]ImH, where Im = imidazole) and KP1019 ([*trans*-RuCl_4_(In)_2_]InH, where In = indazole) have been on clinical trials.^5^ Ru(iii) complexes are paramagnetic, but diamagnetic low-spin 4d^6^ Ru(ii) complexes, can in principle, be studied by ^99^Ru and ^101^Ru NMR, Table 1. Their natural abundances are reasonable (12.76% and 17.06%, respectively), and although their gyromagnetic ratios are relatively low, they should be detectable with similar sensitivities as ^13^C. However, both are quadrupolar with *I* = <svg xmlns="http://www.w3.org/2000/svg" version="1.0" width="18.545455pt" height="16.000000pt" viewBox="0 0 18.545455 16.000000" preserveAspectRatio="xMidYMid meet"><metadata> Created by potrace 1.16, written by Peter Selinger 2001-2019 </metadata><g transform="translate(1.000000,15.000000) scale(0.015909,-0.015909)" fill="currentColor" stroke="none"><path d="M0 760 l0 -120 160 0 160 0 0 -80 0 -80 -120 0 -120 0 0 40 0 40 -40 0 -40 0 0 -40 0 -40 40 0 40 0 0 -40 0 -40 120 0 120 0 0 40 0 40 40 0 40 0 0 80 0 80 -40 0 -40 0 0 40 0 40 -120 0 -120 0 0 40 0 40 160 0 160 0 0 40 0 40 -200 0 -200 0 0 -120z M720 840 l0 -40 -40 0 -40 0 0 -80 0 -80 -40 0 -40 0 0 -80 0 -80 -40 0 -40 0 0 -80 0 -80 -40 0 -40 0 0 -80 0 -80 -40 0 -40 0 0 -40 0 -40 -40 0 -40 0 0 -40 0 -40 80 0 80 0 0 40 0 40 40 0 40 0 0 80 0 80 40 0 40 0 0 80 0 80 40 0 40 0 0 -40 0 -40 80 0 80 0 0 40 0 40 40 0 40 0 0 -80 0 -80 -40 0 -40 0 0 -40 0 -40 -40 0 -40 0 0 -40 0 -40 -40 0 -40 0 0 -40 0 -40 200 0 200 0 0 40 0 40 -80 0 -80 0 0 40 0 40 40 0 40 0 0 80 0 80 40 0 40 0 0 40 0 40 -40 0 -40 0 0 40 0 40 -120 0 -120 0 0 -40 0 -40 -40 0 -40 0 0 80 0 80 40 0 40 0 0 80 0 80 40 0 40 0 0 80 0 80 -40 0 -40 0 0 -40z"/></g></svg> ; ^101^Ru has a higher quadrupole moment than ^99^Ru. Both isotopes give rise to broad lines unless in highly symmetrical complexes. The complexes [Ru(NH_3_)_6_]Cl_2_ and K_4_[Ru(CN)_6_] (notably with a temperature coefficient for the chemical shift of >1 ppm K^−1^) give narrow resonances and are useful references.^6^^99^Ru solution studies on such symmetrical complexes are also possible.^7^ For tris-(polypyridyl)Ru(ii) complexes in acetonitrile, a correlation between the ^99^Ru-NMR chemical shifts and the energy of metal-to-ligand charge transfer has been reported.^8^ The chemical shift range for ^99^Ru is >9000 ppm. The ^99^Ru NMR linewidth of 0.15 M [Ru^II^(bipyridine)_3_]PF_6_ in CD_3_CN is 65 Hz, and for other polypyridyl complexes up to 800 Hz, depending on the counter-anion and concentration.^9^ There is medical interest in related complexes as photodynamic agents for the treatment of cancer. TLD-1433, [Ru(4,4′-dimethyl-2,2′-bipyridine)_2_(2-(2′,2′′:5′′,2′′′-terthiophene)-imidazo[4,5-*f*][1,10]phenanthroline)]Cl_2_, an octahedral tris-diimine Ru(ii) complex with two methylated bipyridyl and one phenanthroline derivative as ligands, is activated by green light and currently on clinical trial for treatment of non-muscle invasive bladder cancer (NMIBC), which is refractory to Bacillus Calmette–Guérin (BCG) treatment.^10,11^ Complexes of lower symmetry, such as half-sandwich Ru(ii) arene anticancer complexes give resonances which are usually too broad to observe easily (unpublished data).

**Table 1:** Properties of isotopes of group 8 transition metals which possess a nuclear spin^12^

Metal	Isotope	Abundance (%)	Nuclear spin (I)	Quadrupole moment^a^	Frequency^b^	Receptivity^c^ (Rel. to ^13^C)
Iron	^57^Fe	2.119	1/2	—	12.955	4.25 × 10^−3^

Ruthenium	^99^Ru	12.76	5/2	7.9	16.427	0.85
	^101^Ru	17.06	5/2	45.7	12.750	1.58

Osmium	^187^Os	1.96	1/2	—	9.132	1.43 × 10^−3^
	^189^Os	16.15	3/2	85.6	31.072	2.32

a Electric quadrupole moment in units of fm^2^ (10^−30^ m^2^; 1 barn = 100 fm^2^).

b ^1^H 400 MHz, ^13^C 100 MHz.

c Receptivity at natural abundance relative to ^13^C.

Although both Ru(ii) and Ru(iii) complexes have been widely explored as anticancer agents, similar studies of osmium complexes are more recent.^13–15^ Os(vi) complexes, of general formula [Os{N}(N^N)Cl_3_] (where N^N = 2,2′-bipyridine or a phenanthroline derivative), can attack DNA and induce endoplasmic reticulum stress, probably through protein binding.^16,17^ Half sandwich diamine Os(ii) arene anticancer complexes can also bind to DNA and inhibit DNA synthesis,^18–20^ whereas azopyridine complexes are more inert and have a redox mechanism of action. Particularly potent is the iodido complex [Os(η^6^-*p*-cym)(*N*,*N*-azpy-NMe_2_)I]PF_6_ (FY26; *p*-cym = *para*-cymene, azpy-NMe_2_ = 2-(*p*-((dimethylamino)phenylazo)pyridine), which is *ca.* 49× more active on average than the anticancer drug cisplatin towards a Sanger Institute panel of over 800 cancer cell lines, and induces formation of reactive oxygen species (ROS) in cancer cells.^21,22^ This complex is capable of delaying the growth of HCT-116 human colon cancer xenografts in mice,^23^ and is up to 64× more selective towards A2780 ovarian cancer cells over normal cells when applied synergistically with l-buthionine sulfoximine, which blocks glutathione (γ-l-Glu-l-Cys-Gly, GSH) synthesis.^24^ The complex FY26 is inert to hydrolysis, but undergoes activation in cancer cells by attack on the azo bond by the intracellular thiol GSH, resulting in liberation of the iodido ligand.^25^

Additional current interest in organo-osmium(ii) half sandwich complexes arises from their activity as transfer hydrogenation catalysts. For example, chiral arene (*p*-toluenesulfonyl)-1,2-diphenylethylenediamine (TsDPEN) complexes, [Os(arene)(TsDPEN)], are effective catalysts for the conversion of the natural metabolite pyruvate into either natural l-lactate or unnatural d-lactate depending on the enantiomer of the catalyst used, reactions of which can be achieved even inside cancer cells.^26,27^

Osmium NMR studies are highly challenging, firstly, the natural abundance in the Earth's crust is very low, *ca.* 1 g per 200 tonnes, making it amongst the rarest stable elements in the periodic table.^28^ There are seven stable naturally-occurring osmium isotopes:^29^^184^Os (0.02%), ^186^Os (1.59%), ^187^Os (1.96%), ^188^Os (13.24%), ^189^Os (16.15%), ^190^Os (26.26%), ^192^Os (40.78%), one of which (^186^Os) is a radioisotope with a half-life of 2.011 × 10^15^ years (α-decay), and for practical purposes is considered ‘stable’. Of the stable isotopes, ^187^Os and ^189^Os possess a nuclear spin (*I* = ½ and <svg xmlns="http://www.w3.org/2000/svg" version="1.0" width="18.545455pt" height="16.000000pt" viewBox="0 0 18.545455 16.000000" preserveAspectRatio="xMidYMid meet"><metadata> Created by potrace 1.16, written by Peter Selinger 2001-2019 </metadata><g transform="translate(1.000000,15.000000) scale(0.015909,-0.015909)" fill="currentColor" stroke="none"><path d="M80 840 l0 -40 -40 0 -40 0 0 -40 0 -40 40 0 40 0 0 40 0 40 120 0 120 0 0 -40 0 -40 -80 0 -80 0 0 -40 0 -40 80 0 80 0 0 -80 0 -80 -120 0 -120 0 0 40 0 40 -40 0 -40 0 0 -40 0 -40 40 0 40 0 0 -40 0 -40 120 0 120 0 0 40 0 40 40 0 40 0 0 80 0 80 -40 0 -40 0 0 40 0 40 40 0 40 0 0 40 0 40 -40 0 -40 0 0 40 0 40 -120 0 -120 0 0 -40z M720 840 l0 -40 -40 0 -40 0 0 -80 0 -80 -40 0 -40 0 0 -80 0 -80 -40 0 -40 0 0 -80 0 -80 -40 0 -40 0 0 -80 0 -80 -40 0 -40 0 0 -40 0 -40 -40 0 -40 0 0 -40 0 -40 80 0 80 0 0 40 0 40 40 0 40 0 0 80 0 80 40 0 40 0 0 80 0 80 40 0 40 0 0 -40 0 -40 80 0 80 0 0 40 0 40 40 0 40 0 0 -80 0 -80 -40 0 -40 0 0 -40 0 -40 -40 0 -40 0 0 -40 0 -40 -40 0 -40 0 0 -40 0 -40 200 0 200 0 0 40 0 40 -80 0 -80 0 0 40 0 40 40 0 40 0 0 80 0 80 40 0 40 0 0 40 0 40 -40 0 -40 0 0 40 0 40 -120 0 -120 0 0 -40 0 -40 -40 0 -40 0 0 80 0 80 40 0 40 0 0 80 0 80 40 0 40 0 0 80 0 80 -40 0 -40 0 0 -40z"/></g></svg> , respectively), making them both magnetically receptive.^30^^189^Os has a high quadrupole moment (*Q* = 0.91) and low gyromagnetic ratio (Table 1), making it relatively insensitive to detection (1.43 × 10^−3^ at natural abundance relative to ^13^C), due to quadrupolar relaxation. ^189^Os is therefore unsuitable for most NMR studies, with the exception of highly symmetrical molecules like ^189^OsO_4_, which yields a broad signal.

^187^Os is a radioactive decay product of ^187^Re (half-life of 4.56 × 10^10^ years) and has been studied extensively in the dating of terrestrial and meteoric rocks, and hydrocarbon deposits.^31^ It is an *I* = ½ nucleus capable of yielding sharp NMR peaks over a chemical shift range of >5000 ppm. Bell *et al.* demonstrated that the use of polarisation transfer techniques to observe *J*(^187^Os–^1^H/^31^P) couplings can greatly enhance the sensitivity of ^187^Os detection, with maximum gains of 1300 and 12 000 for ^31^P and ^1^H-detected spectra.^32^ This polarisation transfer approach had been applied also to μ-hydrido and Cp complexes.^32–34^ Bell *et al.* utilised one-bond ^187^Os–^1^H (hydride) couplings of *ca.* 70 Hz, and ^187^Os–^31^P couplings to phosphines of *ca.* 270 Hz, to detect ^187^Os NMR peaks for 37 [Os(arene)X_2_(PR_3_)] complexes. In a previous study on the biphenyl sandwich complex [Os(η^6^-bip)_2_](OTf)_2_, we used ^1^H/^187^Os heteronuclear multiple bond correlation (HMBC) spectroscopy to observe the ^187^Os resonance for a 25 mM solution of the complex *via* the small ^187^Os–^1^H couplings to arene ring protons.^35^

Here we have synthesised the bromido analogue of the iodido complex FY26, [Os(η^6^-*p*-cym)(*N*,*N*-azpy-NMe_2_)Br]PF_6_ (**1**), and determined its X-ray crystal structure. Also we have synthesised on a milligram scale, the ^187^Os isotopically-enriched complex [^187^Os]-**1**, carried out multinuclear NMR and MS studies, and determined the antiproliferative activity of **1** towards human cancer cells. This appears to be the first report of NMR studies on a ^187^Os-enriched complex.

## Results and discussion

### Synthesis and characterisation

**Scheme 1:**
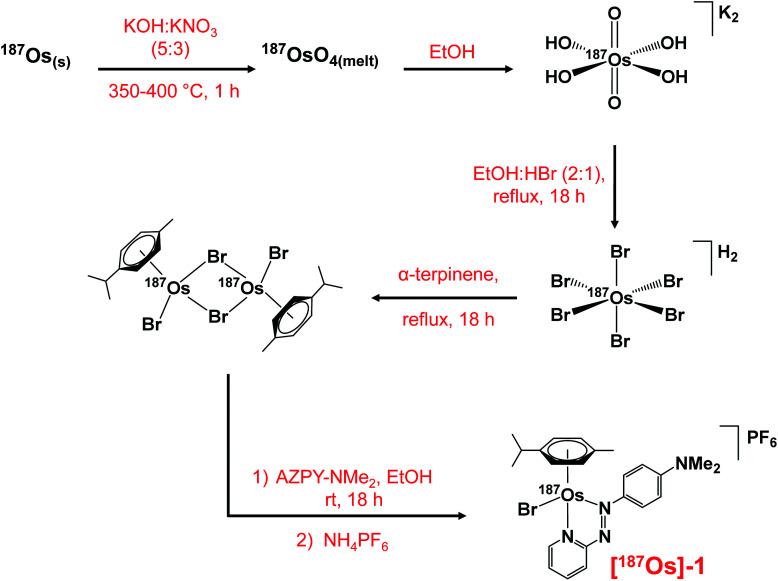
The multi-step synthesis of [^187^Os(η^6^-*p*-cym)(*N*,*N*-azpy-NMe_2_)Br]PF_6_ with ^187^Os isotopic enrichment, starting from ^187^Os metal powder.

The synthesis of the ^187^Os isotopically-enriched complex, [^187^Os(η^6^-*p*-cym)(*N*,*N*-azpy-NMe_2_)Br]PF_6_ ([^187^Os]-**1**), from *ca.* 50 mg metallic osmium-187 (98% enriched) was achieved in five steps based on previously reported methods (Scheme 1), with an overall yield of 41%.^36,37^ The non-enriched complex **1** was synthesised from K_2_[OsO_2_(OH)_4_], also *via* reported methods, and is analogous to the highly active complex [Os(η^6^-*p*-cym)(*N*,*N*-azpy-NMe_2_)I]PF_6_ (FY26),^21^ with bromide replacing iodide as the monodentate ligand.

**Fig. 1:**
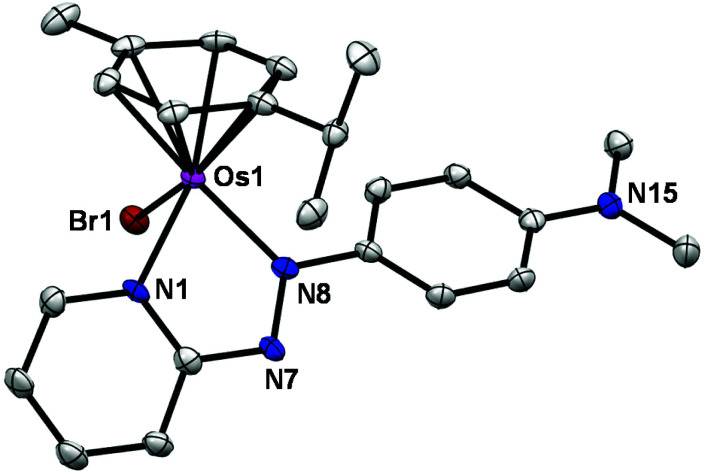
ORTEP diagram for the cation in the X-ray crystal structure of [Os(η^6^-*p*-cym)(azpy-NMe_2_)Br]PF_6_. Ellipsoids are shown at the 50% probability level and all hydrogens, counter ions and solvent molecules have been omitted for clarity.

The X-ray crystal structure of complex **1** shows that it adopts a similar pseudo-octahedral three-legged piano-stool geometry to its analogue, FY26, (Fig. 1, Table S1†).^25^ Osmium(ii) is π-bonded to a *p*-cym ligand, and coordinated to a monodentate bromide and a bidentate azopyridine ligand, which constitute the three legs of the piano-stool. The complex crystallises as a racemate owing to the chiral Os centre, and contains a PF_6_^−^ counter-anion.

### Anticancer activity

Similarly to FY26, complex **1** exhibits potent sub-micromolar antiproliferative activity against A2780 human ovarian cancer cells (IC_50_ = 0.40 ± 0.01 μM, 24 h drug exposure, 72 h recovery) and probably has a similar mechanism of action, inducing formation of reactive oxygen species (ROS) and perturbing the redox balance in cancer cells. The IC_50_ follows an expected trend of activity for change in the monodentate ligand: I > Br > Cl, whereby the iodido complex is the most active and the chlorido complex is the least, due to weakening of the Os–X bond decreasing stability, and hence greater drug deactivation before reaching the target sites (Table S2†). This trend was observed previously for a related bromido Os(ii) arene azopyridine complex and its halido analogues.^38^ The racemic nature of the complex would be expected to have little effect on activity, since previous studies on the separated enantiomers of FY26 showed that the chiral centre does not significantly change the anticancer activity.^39^

### Mass spectrometry

**Fig. 2:**
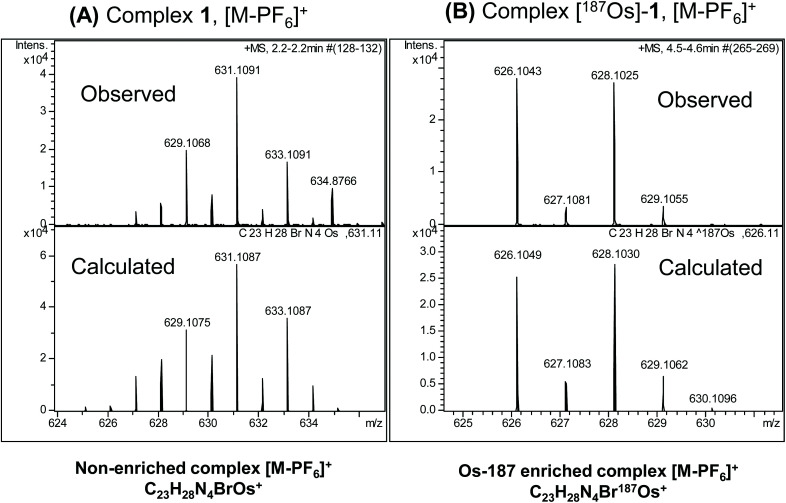
Observed and calculated positive-ion high resolution mass spectra of (A) natural abundance complex **1** ([M − PF_6_]^+^; [C_23_H_28_N_4_BrOs]^+^), and (B) ^187^Os-enriched [^187^Os]-**1** ([M − PF_6_]^+^; [C_23_H_28_N_4_Br^187^Os]^+^). The simplification of the spectrum on enrichment due to the presence of predominantly only one osmium isotope (^187^Os) together with ^79^Br (51%) and ^81^Br (49%) is apparent.

The observed and calculated positive-ion high resolution mass spectra of natural abundance complex **1** ([M − PF_6_]^+^; [C_23_H_28_N_4_BrOs]^+^) and [^187^Os]-**1** ([M − PF_6_]^+^; [C_23_H_28_N_4_Br^187^Os]^+^) are compared in Fig. 2A and B, respectively. The spectrum of enriched [^187^Os]-**1** is greatly simplified due to the presence of predominantly only one osmium isotope (^187^Os) instead of 4 additional isotopes (^188^Os 13.24%, ^189^Os 16.15%, ^190^Os 26.26%, and ^192^Os 40.78%). The presence of ^79^Br (natural abundance 51%) and ^81^Br (49%) isotopes is apparent.

### ^1^H NMR

**Fig. 3:**
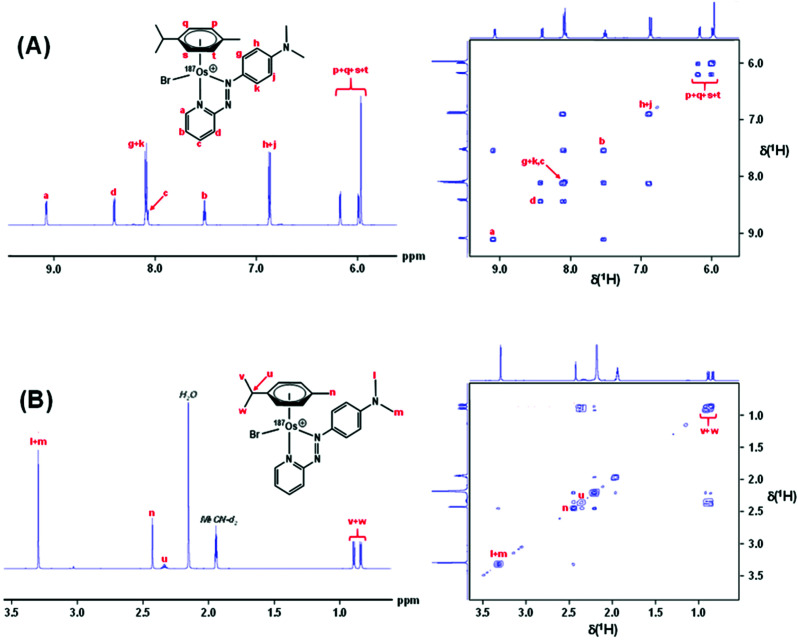
700 MHz ^1^H NMR spectrum of [^187^Os]-**1** (LHS), and 400 MHz 2D ^1^H COSY NMR spectrum of **1** with all ^1^H resonances assigned. (A) The aromatic region, and (B) the aliphatic region. Samples were prepared in MeCN-*d*_3_.

**Fig. 4:**
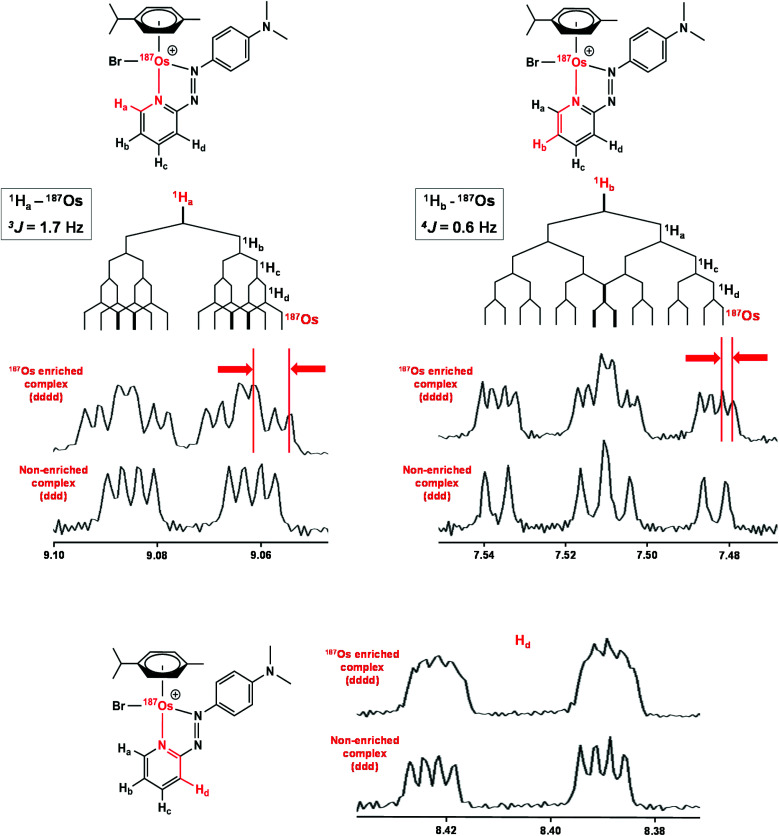
^1^H NMR (250 MHz) spectra of [^187^Os]-**1** compared to natural abundance **1**, with expansions of the resonances for ^1^H_a_, ^1^H_b_, and ^1^H_d_ illustrating their couplings to other protons and ^187^Os. Samples were prepared in MeCN-*d*_3_.

Complexes **1** and [^187^Os]-**1** were prepared in MeCN-*d*_3_ (∼10 mg mL^−1^) and ^1^H COSY NMR spectra were recorded on 400 and 700 MHz instruments (Fig. 3), and the resonances assigned. To resolve coupling interactions between ^187^Os and ^1^H nuclei, 250 MHz ^1^H NMR spectra of the samples were also recorded (Fig. 4). As expected, no ^187^Os–^1^H coupling interactions were observed for **1** due to the low natural abundance of ^187^Os (1.96%). However, the ^1^H spectrum of [^187^Os]-**1** shows the presence of 3- and 4-bond couplings between ^187^Os and ^1^H_a_ (^3^*J* = 1.7 Hz), and ^1^H_b_ (^4^*J* = 0.6 Hz). There was also a small unresolved coupling to ^1^H_d_; the resolution was not sufficient to measure ^4^*J*. The resonance for proton ^1^H_c_ is overlapped with other resonances, although the ^5^*J* value might be expected to be very small compared to the linewidth.

### ^13^C NMR

**Fig. 5:**
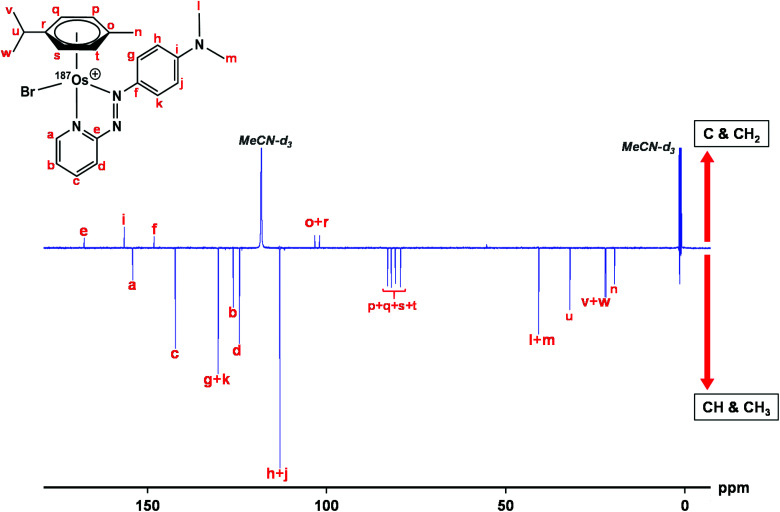
176 Hz ^13^C NMR spectrum of [^187^Os]-**1** using the J-MOD sequence, with ^13^C assignments. The sample was prepared in MeCN-*d*_3_. C and CH_2_ peaks are upright, CH and CH_3_ peaks inverted.

**Fig. 6:**
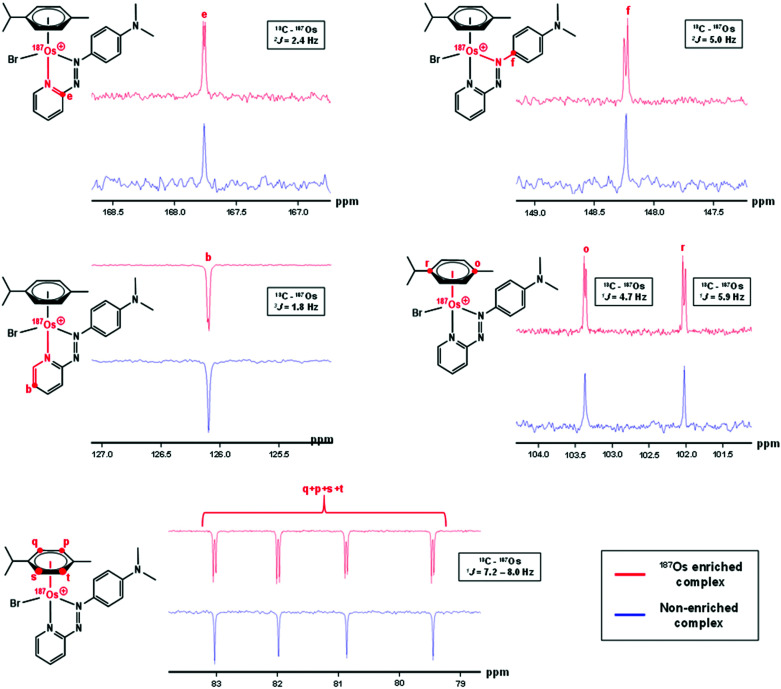
176 MHz ^13^C NMR spectra of [^187^Os]-**1** compared to natural abundance **1** (150 MHz), with expansions of resonances for ^13^C_b_, ^13^C_e_, ^13^C_f_, ^13^C_o_, ^13^C_p_, ^13^C_q_, ^13^C_r_, ^13^C_s_, and ^13^C_t_, which all exhibit couplings to ^187^Os. Samples were prepared in MeCN-*d*_3_.

The samples used for ^1^H NMR studies were also analysed by ^13^C NMR, utilising a J-MOD sequence (Fig. 5). To aid characterisation of the resonances, a ^13^C–^1^H HMQC NMR spectrum was recorded (Fig. S1†), and all 23 ^13^C resonances were observed and characterised in the spectrum. Furthermore, expansions of some resonances reveal distinct ^187^Os–^13^C couplings for [^187^Os]-**1** (Fig. 6). Short-range couplings are observed between ^187^Os and ^13^C_p_, ^13^C_q_, ^13^C_s_, and ^13^C_t_ (^1^*J* = 7.2–8.0 Hz), which comprise the four CH carbons of the arene ring of the *p*-cym ligand, and between ^187^Os and the two tertiary carbons of the ring, ^13^C_o_ and ^13^C_r_ (^1^*J* = 4.7 and 5.9 Hz, respectively). Furthermore, longer range couplings are observed between ^187^Os and ^13^C_e_ and ^13^C_f_ (^2^*J* = 2.4 and 5.0 Hz, respectively), and between ^187^Os and ^13^C_b_ (^3^*J* = 1.8 Hz), which comprise ^13^C resonances of the bidentate azpy ligand. Table 2 lists all the observed ^187^Os–^1^H and ^187^Os–^13^C couplings.

**Table 2:** ^187^Os *J*-couplings to ^1^H and ^13^C determined by ^1^H and ^13^C NMR

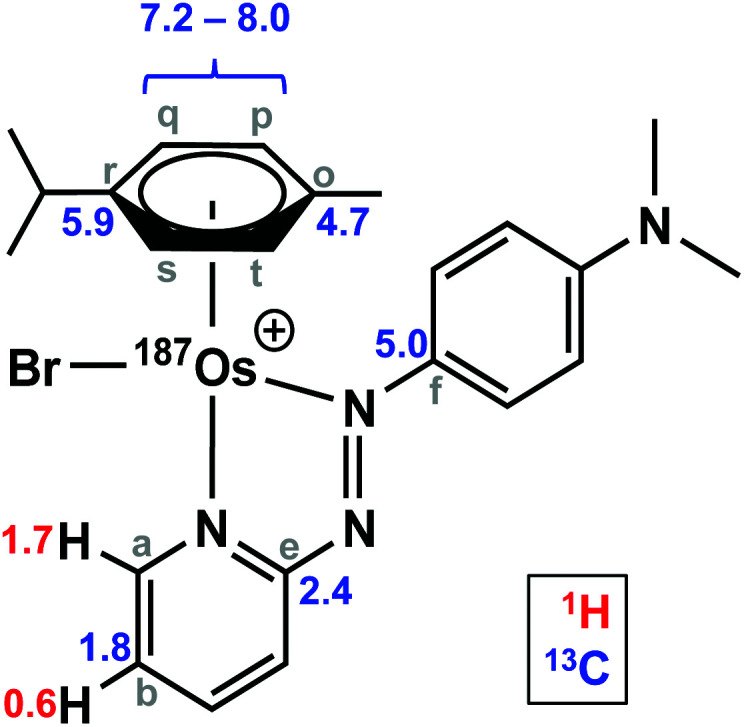	
Coupling	*J*^a^ (Hz)
^3^*J*(^1^H_a_ – ^187^Os)	1.7
^4^*J*(^1^H_b_ – ^187^Os)	0.6
^1^*J*(^13^C_o_ – ^187^Os)	4.7
^1^*J*(^13^C_r_ – ^187^Os)	5.9
^1^*J*(^13^C_q,p,s,t_ – ^187^Os)	7.2–8.0
^2^*J*(^13^C_e_ – ^187^Os)	2.4
^2^*J*(^13^C_f_ – ^187^Os)	5.0
^3^*J*(^13^C_b_ – ^187^Os)	1.8

a The signs of the coupling constants have not been determined.

### ^1^H–^187^Os HMBC

**Fig. 7:**
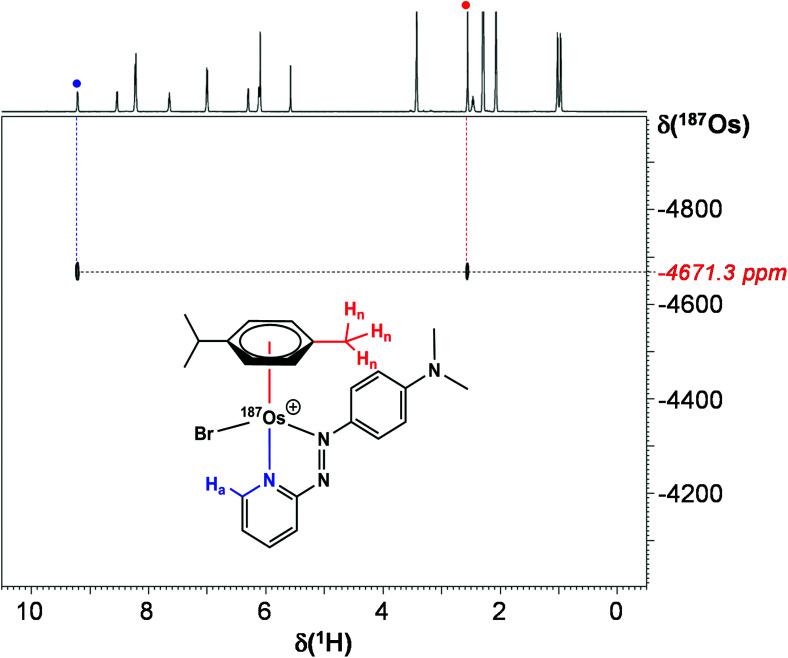
2D ^1^H–^187^Os HMBC NMR spectrum of [^187^Os]-**1** in MeCN-*d*_3_.

**Fig. 8:**
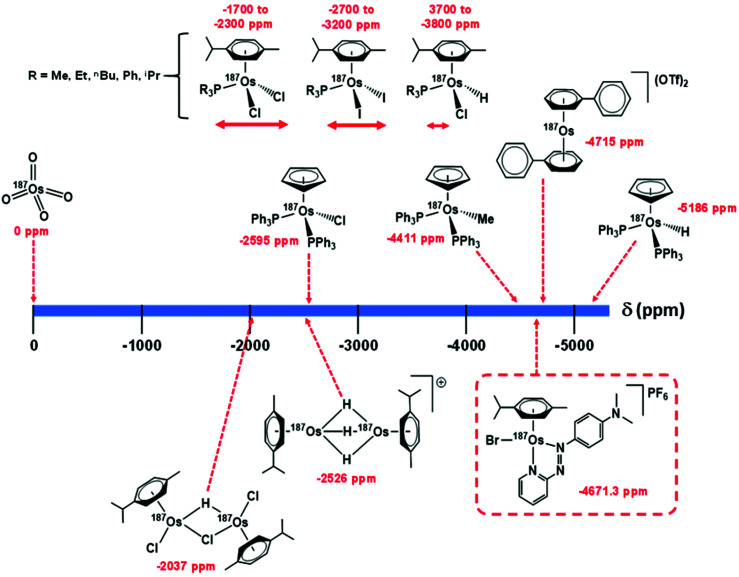
Comparison of ^187^Os chemical shifts for [^187^Os]-**1** in comparison with values for arene and cyclopentadienyl Os(ii) complexes in the literature.^33–35,40^ Shifts are either referenced directly to OsO_4_ or indirectly from the proton frequency of TMS.

Utilising ^1^H–^187^Os HMBC, we were able to observe the ^187^Os resonance of [^187^Os]-**1** and determine its chemical shift relative to OsO_4_. The resonance was observed at −4671.3 ppm through correlation with aromatic proton H_a_ (pyridyl ring), and aliphatic protons from the arene ligand, H_n_ (Fig. 7). Attempts to observe directly the ^187^Os resonance of [^187^Os]-**1** were unsuccessful (Fig. S2†). The very low gyromagnetic ratio of ^187^Os makes direct observation difficult, despite the complex being isotopically enriched. Fig. 8 shows a comparison of ^187^Os chemical shifts for various organo-osmium complexes with arene and cyclopentadienyl ligands. The comparatively low chemical shift of the (high-field-shifted) ^187^Os resonance of [^187^Os]-**1**, similar to a bis-biphenyl arene Os(ii) sandwich complex, suggests a highly shielded ^187^Os nucleus, perhaps surprising on account of the presence of the strong π-acceptor azopyridine ligand, although the comparator complexes mostly contain phosphines, or hydride ligands. Future work is required to elucidate the relationship between the electron densities in these complexes and the ^187^Os chemical shifts, *e.g.* DFT calculations.

### Heteronuclear NMR for metallodrugs

Most NMR spectroscopic studies of metallodrugs involve nuclei which are the most sensitive to detection. Only a few NMR nuclei offer the sensitivity for studies at pharmacological concentrations. These include ^1^H, ^19^F, ^31^P, and ^13^C and ^15^N with enrichment and detection by polarisation transfer methods. Studies of NMR-active isotopes of metals present significant challenges.^41–43^

Sensitivity is a function of the size of the gyromagnetic ratio (*γ*, proportional to the frequency of detection of resonances), the natural abundance, and the applied magnetic field (*B*_0_). NMR signal intensity is proportional to *B*^3/2^_0_*γ*^5/2^. In general, as nuclei become heavier, they have smaller gyromagnetic ratios and they become less sensitive to detection. The detection sensitivity of low abundance nuclei such as ^13^C and ^15^N, which are often coupled to more sensitive ^1^H nuclei, can be greatly enhanced by use of spin-polarisation-transfer pulse sequences such as heteronuclear single quantum coherence (HSQC), *e.g.* for ^15^N by up to a factor of 306 (|γ^1^H|/|γ^15^N|)^5/2^.

All elements in the periodic table from atomic number 1 to 103 have at least one isotope which possesses nuclear spin.^29,44^ Radioactivity can be a problem for some elements, although shielding by glass is adequate for tritium (^3^H), which has a higher gyromagnetic ratio than ^1^H and is 1.21× more sensitive to detection. A more common problem is that nuclei with spin quantum numbers >½ (quadrupolar nuclei) in environments that are not symmetrical, usually suffer line broadening *via* interaction with electric field gradients. For nuclei with high quadrupole moments (*e.g.*^197^Au), this can lead to extreme broadening making resonances very difficult, and sometimes almost impossible to detect. Line broadening can also be severe for paramagnetic metal complexes, especially complexes where the unpaired spins have long electron spin relaxation times (those detectable by EPR at ambient temperature), and when there are exchange processes which occur at intermediate rates on the NMR timescale. The resonances of heavier nuclei can be broadened by chemical shift anisotropy relaxation mechanisms (*e.g.*^195^Pt in square-planar complexes), which also broadens ^195^Pt satellites in ^1^H NMR spectra of Pt(ii) ligands. Such broadening is proportional to *B*^2^_0_ so can make such satellites very broad at high frequencies (*e.g.* at 600 MHz *versus* 300 MHz).^45^

There is also current pharmacological interest in the group 8 metal iron. Ferrocene in particular shows promise as a fragment for conjugation in drug design.^46,47^ Ferroquine is on clinical trial as an antimalarial drug,^48^ and ferrocifens (tamoxifen derivatives) have promising anticancer activity especially towards breast cancer cells possessing the estrogen receptor (ER+).^49^^57^Fe is an *I* = ½ nucleus, but the abundance is low (2.1%), as is the gyromagnetic ratio, Table 1. With polarisation transfer from ^1^H, utilising the small two-bond Cp (C–H) ^1^H–^57^Fe coupling of *ca.* 0.5 Hz, ^57^Fe resonances can readily be detected for concentrated solutions of ferrocene (*ca.* 0.4 M).^50^^57^Fe chemical shifts have been used to study the donor properties of amino groups on coordinated Cp rings.^51^^57^Fe isotopic enrichment (to *ca.* 90%) allows detection at millimolar concentrations, *e.g.* for haem proteins.^52^ Enrichment combined with polarisation-transfer methods should allow detection at pharmacological concentrations of relevance to metallodrug activity. However, oxidation to paramagnetic 3d^5^ Fe(iii) is likely to lead to severe broadening and loss of ^57^Fe resonances.

## Conclusions

NMR is a versatile technique for the study of metallodrugs in both the solid state and solution. Here we have focussed on solution studies, which can not only provide structural information, but also insights into both the thermodynamics and kinetics of ligand exchange reactions. Such information is vital for identifying pharmacologically active species. The common spin-½ nuclei ^1^H, ^19^F and ^31^P are sensitive enough for NMR studies at physiologically relevant concentrations (often sub-millimolar), and so too are ^13^C and ^15^N in isotopically-enriched compounds, especially when polarisation transfer methods such as HSQC are used. In contrast, *I* > ½ (quadrupolar) nuclei usually give rise to broad peaks which limits their usefulness at low concentrations.

Platinum anticancer drugs are now the most widely used drugs in cancer chemotherapy, and NMR studies of the 33.4% abundant *I* = ½ isotope ^195^Pt have proved useful for studies of the activation of cisplatin and related drugs by hydrolysis and subsequent attack of DNA target sites, as well as the reduction of Pt(iv) prodrugs to Pt(ii).^4,41,43,53^ Currently, there is much interest in the discovery of active anticancer complexes of other transition metals, and we have highlighted here the potential for group 8 of the periodic table, iron, ruthenium, and osmium. Complexes of ruthenium have already entered clinical trials,^54^ and one is in clinical trials as a photosensitizer for cancer treatment.^10^ However, ruthenium has only quadrupolar nuclei and direct studies of these drugs by Ru NMR are difficult. The heavier congener, osmium, on the other hand has a useful *I* = ½ isotope ^187^Os, although with a low natural abundance and low gyromagnetic ratio (Table 1).

We have shown here that isotopic enrichment of a potent anticancer complex [^187^Os(η^6^-*p*-cym)(*N*,*N*-azpy-NMe_2_)Br]PF_6_ to >98% ^187^Os allows facile detection of its ^187^Os resonance NMR using 2D ^1^H–^187^Os HMBC, and accompanying ^1^H and ^13^C NMR studies allow detection for the first time of ^1^*J*, ^2^*J*, ^3^*J* and ^4^*J* coupling constants for both the arene ring and the chelated azopyridine ligand, ranging from 0.6–8 Hz. However, despite isotopic enrichment, direct observation of the ^187^Os resonance (without the use of spin-polarisation-transfer techniques) was not achievable due to its very low gyromagnetic ratio. The synthesis of the enriched complex was achieved in good yield over 5 steps from metallic ^187^Os, the first step of which involved conversion to ^187^OsO_4_, a highly toxic volatile compound generated in a KOH/KNO_3_ melt at >350 °C. The mechanism of anticancer activity of this class of organo-osmium arene anticancer complexes appears to be different from that of platinum anticancer drugs, and further studies using such ^187^Os-enriched complexes are likely to provide new insight into their reactions in biological media and the nature of their target sites.

## Experimental

### Materials

#### Chemicals

^187^Os powder was the kind gift of Dr Dimitrios Bessas (ESRF, Grenoble). K_2_[OsO_2_(OH)_4_] and *N*,*N*-dimethyl-4-(2-pyridylazo)aniline were purchased from Alfa Aesar, and α-terpinene and ammonium hexafluorophosphate were purchased from Sigma-Aldrich. All other general reagents (KOH, KNO_3_, and HBr), and organic solvents were purchased from commercial suppliers and used as received. Deionised water for synthesis was produced using a Millipore Elix 5 water purification system.

#### Cell culture

DMEM cell culture media, penicillin/streptomycin mixture, foetal bovine serum, l-glutamine, and trypsin were all purchased from PAA Laboratories GmbH. DMEM was supplemented with foetal bovine serum (50 mL), penicillin/streptomycin mixture (5 mL), and l-glutamine (5 mL). The A2780 cell line was purchased from the European Collection of Cell Cultures.

### Synthesis

#### [^187^Os(η^6^-*p*-cym)Br_2_]_2_

Powdered osmium-187 (50.3 mg, 0.267 mmol), KOH (0.5 g) and KNO_3_ (0.3 g) were mixed in an alumina crucible and heated to 350–400 °C, yielding a brown melt. After cooling to ambient temperature the solids were dissolved in H_2_O (10 mL). EtOH (20 mL) was added and a pink-purple precipitate formed. The solvents were removed by decantation and the precipitate washed with EtOH : H_2_O (3 : 1, v/v, 3 × 15 mL). The clean precipitate was mixed with EtOH (10 mL), HBr (48%, v/v, 5 mL) and heated under reflux for 18 h. α-Terpinene (501 mg, 3.678 mmol) was added and the mixture was refluxed for a further 18 h. The product was extracted with CH_2_Cl_2_ (25 mL) and washed with H_2_O (3 × 20 mL). The solution was concentrated to ∼3 mL under reduced pressure and a brown precipitate formed, which was placed in a freezer overnight (253 K). The precipitate was collected *via* vacuum filtration and washed with EtOH (5 mL) and Et_2_O (5 mL). Yield (53.1 mg, 41%). No analysis was conducted at this stage to avoid loss of valuable ^187^Os. *Note*: Extreme care must be taken with the handling of the OsO_4_ intermediate (Scheme 1).

Natural abundance [Os(η^6^-*p*-cym)Br_2_]_2_ was synthesised from K_2_[OsO_2_(OH)_4_] using a previously reported method.^37^

#### [^187^Os(η^6^-*p*-cym)(*N*,*N*-azpy-NMe_2_)Br]PF_6_

[^187^Os(η^6^-*p*-cym)Br_2_]_2_ (12.6 mg, 13.10 μmol) was dissolved in EtOH (10 mL), and a solution of *N*,*N*-dimethyl-4-(2-pyridylazo)aniline (6.2 mg, 27.322 μmol) in EtOH (5 mL) was added drop-wise to the stirred mixture. The mixture was stirred at 313 K for 2 h, then NH_4_PF_6_ (21.2 mg, 0.130 mmol) was added. The mixture was concentrated to ∼2 mL under reduced pressure and placed in a freezer overnight (253 K). A dark blue precipitate formed, which was collected *via* vacuum filtration and washed with ice-cold EtOH (1 mL) and Et_2_O (5 mL). The product was dried in a vacuum desiccator overnight. Yield: (18.6 mg, 92%). ^1^H NMR 250 MHz (CD_3_CN): *δ* 9.07 (dddd, 1H, *J* = 5.8, 1.6, 1.7, 1.0 Hz), 8.41 (m, 1H), 8.05–8.13 (m, 3H), 7.51 (dddd, 1H, *J* = 7.5, 5.9, 1.4, 0.6 Hz), 6.84–6.90 (m, 2H), 6.16–6.18 (m, 1H), 5.95–6.00 (m, 3H), 3.30 (s, 6H), 2.43 (s, 3H), 2.34 (sept., 1H, *J* = 6.9 Hz), 0.90 (d, 3H, *J* = 6.9 Hz), 0.85 (d, 3H, *J* = 6.9 Hz). ^13^C NMR 176 MHz (CD_3_CN): *δ* 167.75 (*C*), 156.56 (*C*), 154.21 (*C*H), 148.23 (*C*), 142.31 (*C*H), 130.33 (*C*H), 126.09 (*C*H), 124.41 (*C*H), 113.10 (*C*H), 103.36 (*C*), 102.02 (*C*), 83.03 (*C*H), 81.98 (*C*H), 80.87 (*C*H), 79.45 (*C*H), 40.83 (*C*H_3_), 32.08 (*C*H), 22.27 (*C*H_3_), 22.06 (*C*H_3_), 19.64 (*C*H_3_). ESI-MS calculated for C_23_H_28_BrN_4_^187^Os^+^: *m*/*z* 626.1049. Found: 626.1043.

### Natural abundance [Os(η^6^-*p*-cym)(*N*,*N*-azpy-NMe_2_)Br]PF_6_

Synthesised using the same procedure as above with; [Os(η^6^-*p*-cym)Br_2_]_2_ (58.6 mg, 60.548 μmol), *N*,*N*-dimethyl-4-(2-pyridylazo)aniline (30.1 mg, 133.120 μmol), and NH_4_PF_6_ (98.6 mg, 0.605 mmol). Yield: (79.9 mg, 85%). ^1^H NMR 250 MHz (CD_3_CN): *δ* 9.07 (ddd, 1H, *J* = 5.8, 1.4, 0.6 Hz), 8.41 (ddd, 1H, *J* = 8.2, 1.3, 0.6 Hz), 8.05–8.13 (m, 3H), 7.51 (ddd, 1H, *J* = 7.5, 5.9, 1.4 Hz), 6.84–6.90 (m, 2H), 6.16–6.18 (m, 1H), 5.96–6.00 (m, 3H), 3.30 (s, 6H), 2.43 (s, 3H), 2.34 (sept., 1H, *J* = 6.9 Hz), 0.90 (d, 3H, *J* = 6.9 Hz), 0.85 (d, 3H, *J* = 6.9 Hz). ^13^C NMR 150 MHz (CD_3_CN): *δ* 167.75 (*C*), 156.56 (*C*), 154.20 (*C*H), 148.23 (*C*), 142.31 (*C*H), 130.33 (*C*H), 126.09 (*C*H), 124.41 (*C*H), 113.10 (*C*H), 103.36 (*C*), 102.01 (*C*), 83.02 (*C*H), 81.98 (*C*H), 80.86 (*C*H), 79.44 (*C*H), 40.83 (*C*H_3_), 32.08 (*C*H), 22.27 (*C*H_3_), 22.06 (*C*H_3_), 19.63 (*C*H_3_). ESI-MS calculated for C_23_H_28_BrN_4_Os^+^: *m*/*z* 631.1087. Found: 631.1091. CHN analysis: Found: C, 35.37%; H, 3.44%; N, 7.37%. Calculated for C_23_H_28_BrF_6_N_4_OsP: C, 35.62%; H, 3.64%; N, 7.22%.

### X-ray crystal structure

A single crystal of complex **1** was grown by slow evaporation of a methanolic solution (∼3 mg mL^−1^), and its molecular structure was determined by X-ray crystallography (Fig. 1). Diffraction data were collected on an Oxford Diffraction Gemini four-circle system with a Ruby CCD area detector. The structure was refined by full-matrix least squares against *F*^2^ using SHELXL 97 and solved by direct methods using SHELXS(TREF) with additional light atoms found by Fourier methods.^55^ Hydrogen atoms were added at calculated positions and refined using a riding model. Anisotropic displacement parameters were used for all non-H atoms; H-atoms were given an isotropic displacement parameter equal to 1.2 (or 1.5 for methyl and NH H-atoms) times the equivalent isotropic displacement parameter of the atom to which they are attached. The data were processed by the modelling program Mercury 1.4.1. X-ray crystallographic data for complex **1** have been deposited in the Cambridge Crystallographic Data Centre under the accession number CCDC 2067332.†

### Mass spectrometry

Electrospray mass spectra were obtained using a Bruker MaXis UHR-ESI-TOF instrument. Samples of **1** and [^187^Os]-**1** were prepared in methanol and analysed in positive ion mode (500–1000 *m*/*z*).

### NMR spectroscopy

^1^H and ^13^C NMR spectra were acquired in 5 mm NMR tubes at 298 K on Bruker AV-250, AV-400, AV-600 or AV-700 spectrometers in MeCN-*d*_3_. Data processing was carried out using TOPSPIN version 2.1 (Bruker UK Ltd). ^1^H NMR chemical shifts were internally referenced to TMS *via* the residual solvent peak: acetonitrile (*δ* = 1.94 ppm).^56^ Likewise for ^13^C NMR chemical shifts: acetonitrile (*δ* = 1.32 ppm). 1D ^1^H NMR spectra were recorded using standard pulse sequences and 1D ^13^C NMR spectra were recorded using a J-MOD pulse sequence. Typically, ^1^H data were acquired with 16 transients into 32k data points over a spectral width of 14 ppm, and ^13^C data with 8192 transients into 64k data points over a spectral width of 220 ppm.

^1^H–^187^Os HMBC NMR spectroscopy was carried out with MeCN-*d*_3_ as solvent at 298 K on an Advance III 600 (^1^H = 600.13 MHz, ^187^Os = 13.69 MHz) spectrometer equipped with a 5 mm triple resonance broadband inverse (TBI-Lr) ^1^H/^31^P/BB probe with *z*-field gradients. The 90° pulse length for ^187^Os was 11 μs. The ^187^Os resonance frequency was roughly estimated from a series of ^1^H–^187^Os HMBC experiments carried out with incremented values of the carrier frequency. In order to check for the absence of folding in the F1 dimension, two gradient-enhanced HMBC spectra were recorded with different settings of the ^187^Os spectral width and offset (SW = 1000 ppm O1P = −3800 and −4500 respectively). The second, shown in Fig. 7, was recorded with the following experimental conditions: 128 transients collected for each FID, 32 increments, 1/2*J* delay set to 0.1 s, recycling delay 2.25 s; three sine-shaped gradients pulses of 1 ms duration in the intensity ratio of 60 : 20 : 41.83 for coherence (echo type) selection; sine bell multiplication in both dimensions and zero filling to 1024 data points in the F1 dimension was applied before Fourier transform followed by magnitude calculation. The reference frequency for the ^187^Os chemical shift was calculated from the proton frequency of internal TMS, using a conversion factor *Ξ* = 2.282331.

^187^Os NMR spectroscopy was carried out using MeCN-*d*_3_ as solventat 298 K on an Avance III 600 (^1^H = 600.13 MHz, ^187^Os = 13.69 MHz) spectrometer equipped with a 5 mm triple resonance broadband inverse (TBI-Lr) ^1^H/^31^P/BB probe with *z*-field gradients. The 90° pulse length for osmium was 11 μs. A series of experiments was carried out with incremented values of the carrier frequency in order to scan the full 10 000 ppm scale (SW = 1000 ppm O1P = from 4500 to −4500). A standard 1D sequence with power gated decoupling was used with 256 scans, recycling delay 4 s.

### Cytotoxicity assay

A stock solution of **1** was prepared in cell culture medium (5% DMSO). Approximately 5000 A2780 human ovarian cancer cells were seeded per well in 96-well plates. The cells were pre-incubated in drug-free media at 37 °C for 48 h before adding different concentrations of **1**. Cells were exposed to **1** for 24 h at 37 °C and then allowed to recover for 72 h in a drug-free medium at 37 °C. Then the supernatants were removed by suction and the cells washed with PBS. The cells were The SRB assay was used to determine cell viability. Absorbance measurements of the solubilised dye (on a BioRad iMark microplate reader using a 470 nm filter) allowed the determination of viable treated cells compared to untreated controls. IC_50_ values (the concentration at which 50% cell death occurs) were determined as triplicates of duplicates for each complex. ICP-OES (PerkinElmer Optima 5300 DV instrument) was used to determine [Os] of the stock solution of **1**, and the IC_50_ value was corrected for ICP factor.

## Conflicts of interest

There are no conflicts of interest.

## Supplementary Material

DT-050-D1DT02213J-s001

DT-050-D1DT02213J-s002
