# Supplementary material for: NMR studies of group 8 metallodrugs: 187Os-enriched organo-osmium half-sandwich anticancer complex
Source: Dalton Trans. 2021 Aug 24;50(37):12970–81. doi: 10.1039/d1dt02213j (PMC8477448; doi:10.1039/d1dt02213j)
Supplement: DT-050-D1DT02213J-s001 [file DT-050-D1DT02213J-s001.pdf]

Electronic Supplementary Material (ESI) for Dalton Transactions.
This journal is © The Royal Society of Chemistry 2021

NMR studies of group 8 metallodrugs: ^{187}Os-enriched organo-osmium half-sandwich anticancer complex

Russell J. Needham, Ivan Prokes, Abraha Habtemariam, Isolda Romero-Canelón,[#] Guy J. Clarkson, Peter J. Sadler[†]

Department of Chemistry, University of Warwick, Gibbet Hill Road, Coventry CV4 7AL, UK. Email: P.J.Sadler@warwick.ac.uk

[#]Current address: School of Pharmacy, Institute of Clinical Sciences, University of Birmingham, Birmingham B15 2TT, UK

Supporting Information

Tables S1-S2 **p S2**

Figures S1-S2 **p S3, S4**

Table S1: X-ray crystal structure parameters

Parameter	Complex 1
Formula	$C_{23}H_{28}BrF_6N_4OsP$
Molar mass /g mol^{-1}	775.57
Density /mg m^{-3}	1.998
Crystal system	Triclinic
Crystal dimensions /mm	0.60 x 0.40 x 0.14
Space group	P-1
Crystal character	Purple block
a/Å	8.3424(2)
b/Å	12.5262(4)
c/Å	12.6754(3)
α/deg	85.480(2)
β/deg	79.486(2)
γ/deg	82.407(2)
T/K	100(2)
Z	2
$R[F> 4\sigma(F)]$	0.0358
R_w	0.0886
GOF	1.038
Δρ max and min /eÅ^{-3}	2.816 & -2.520

Table S2: A2780 human ovarian cancer cytotoxicity of complexes [Os(η^6-*p*-cym)(*N,N*-Azpy-NMe_2)X]PF_6 for a 24 h drug exposure with 72 h recovery time. X = Cl, Br, I.

Complex	IC_{50} (μM)
1-Cl[a]	1.8±0.1
1-Br	0.40±0.01
1-I[a]	0.18±0.01

[a]Taken from reference[29]

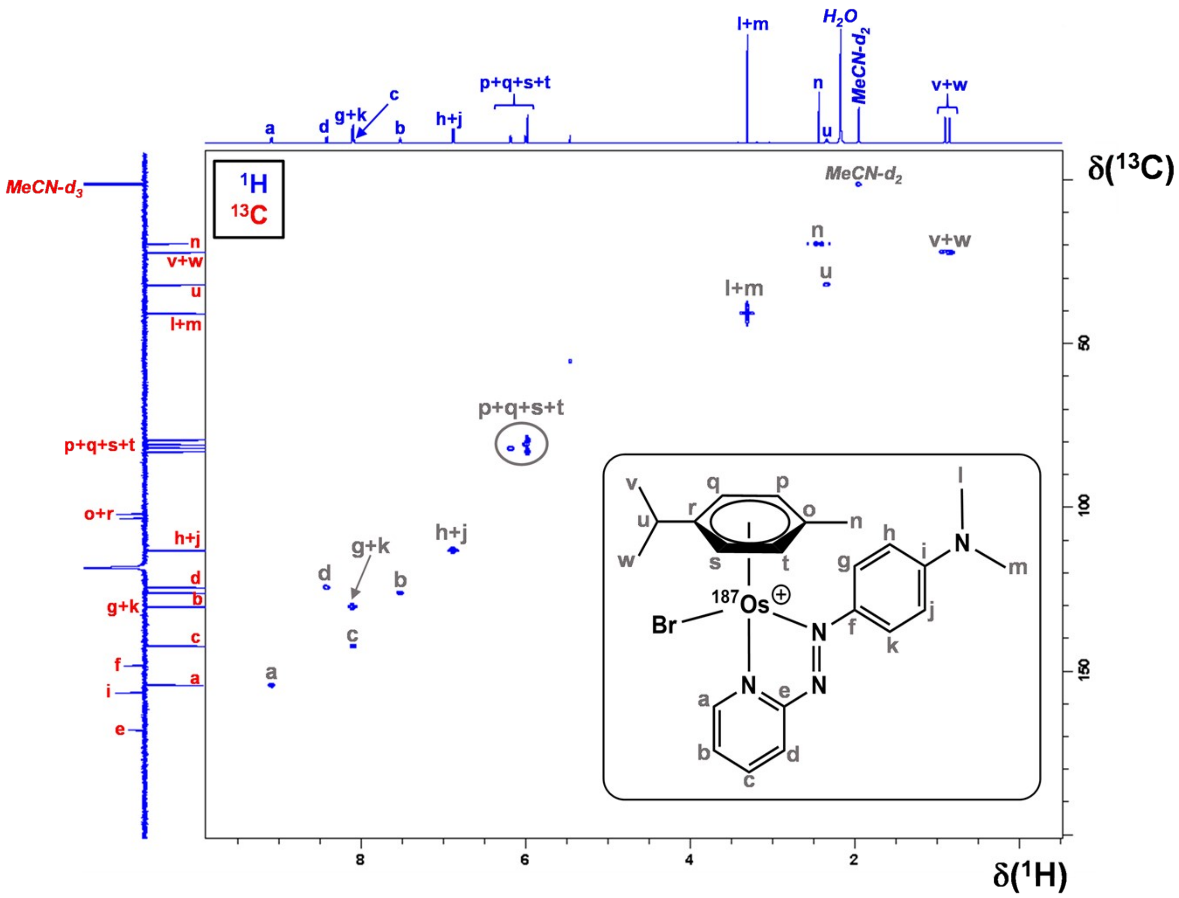

Figure S1: ^{13}C – ^{1}H HMQC 2D NMR spectrum of complex **1** in MeCN-d_3. Channel F1 ^{1}H, 600 MHz, channel F2 ^{13}C, 150 MHz.

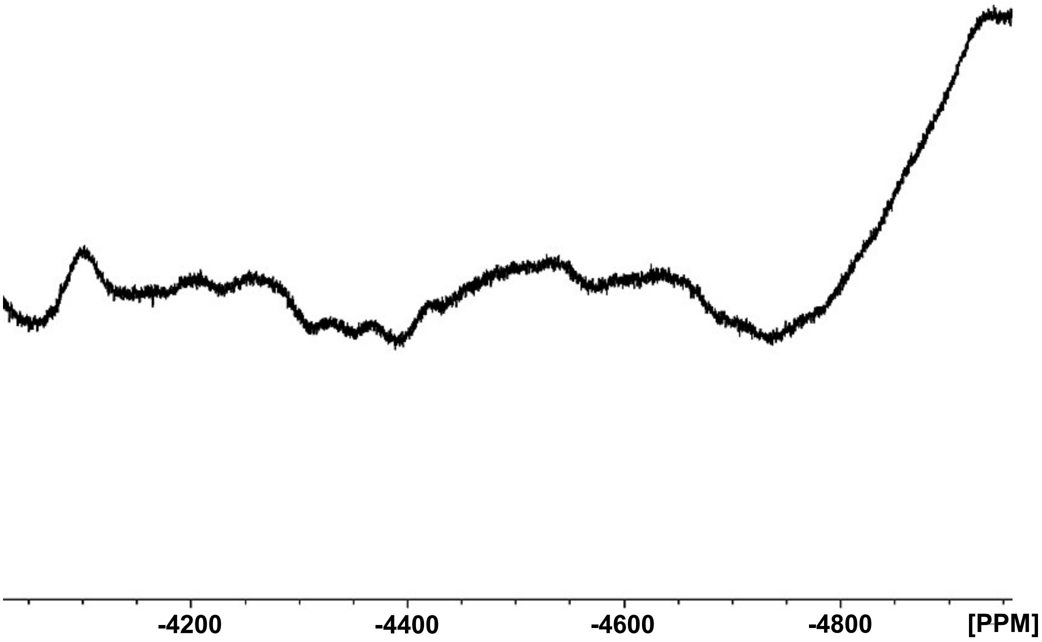

Figure S2: Attempt on direct ^{187}Os NMR detection for [^{187}Os]-**1** (13.69 MHz). Only baseline noise is present.
